# Activation of J77A.1 Macrophages by Three Phospholipases A_2_ Isolated from *Bothrops atrox* Snake Venom

**DOI:** 10.1155/2014/683123

**Published:** 2014-01-27

**Authors:** Juliana L. Furtado, George A. Oliveira, Adriana S. Pontes, Sulamita da S. Setúbal, Caroline V. Xavier, Fabianne Lacouth-Silva, Beatriz F. Lima, Kayena D. Zaqueo, Anderson M. Kayano, Leonardo A. Calderon, Rodrigo G. Stábeli, Andreimar M. Soares, Juliana P. Zuliani

**Affiliations:** ^1^Laboratório de Imunofarmacologia Aplicada à Saúde, Fundação Oswaldo Cruz, FIOCRUZ-Rondônia, Rua da Beira, 7671 Br364, Km 3.5, 76812-245 Porto Velho, RO, Brazil; ^2^Centro de Biomoléculas Aplicadas à Saúde, FIOCRUZ-Rondônia e Departamento de Medicina, Universidade Federal de Rondônia (UNIR), Campus BR 364, Km 9.5, 76801-059 Porto Velho, RO, Brazil

## Abstract

In the present study, we investigated the *in vitro* effects of two basic myotoxic phospholipases A_2_ (PLA_2_), BaTX-I, a catalytically inactive Lys-49 variant, and BaTX-II, a catalytically active Asp-49, and of one acidic myotoxic PLA_2_, BaPLA_2_, a catalytically active Asp-49, isolated from *Bothrops atrox* snake venom, on the activation of J774A.1 macrophages. At noncytotoxic concentrations, the toxins did not affect the adhesion of the macrophages, nor their ability to detach. The data obtained showed that only BaTX-I stimulated complement receptor-mediated phagocytosis. However, BaTX-I, BaTX-II, and BaPLA_2_ induced the release of the superoxide anion by J774A.1 macrophages. Additionally, only BaTX-I raised the lysosomal volume of macrophages after 15 min of incubation. After 30 min, all the phospholipases increased this parameter, which was not observed within 60 min. Moreover, BaTX-I, BaTX-II, and BaPLA_2_ increased the number of lipid bodies on macrophages submitted to phagocytosis and not submitted to phagocytosis. However, BaTX-II and BaPLA_2_ induced the release of TNF-**α** by J774A.1 macrophages. Taken together, the data show that, despite differences in enzymatic activity, the three toxins induced inflammatory events and whether the enzyme is acidic or basic does not seem to contribute to these effects.

## 1. Introduction

*Bothrops atrox* is responsible for most cases of envenoming in the Northern region of Brazil [1]. Its venom contains, among others, proteins serine proteinases, metalloproteinases, L-amino acid oxidases, C-type lectin-like enzymes, and phospholipases A_2_. Phospholipases A_2_ (PLA_2_; EC 3.1.1.4) belong to a diverse super family of lipolytic enzymes that catalyse the hydrolysis of the sn-2 ester bond of membrane glycerophospholipids to produce free fatty acids and lysophospholipids. These enzymes can differ in structure, molecular weight, substrate specificity, requirement for Ca^2+^, cell localization, and mechanism of action [2]. PLA_2_s are classified into five major families: extracellular or secretory PLA_2_s (sPLA_2_), intracellular cytosolic PLA_2_s (cPLA_2_), Ca^2+^-independent PLA_2_s (iPLA_2_), PAF acetylhydrolases, and lysossomal PLAs (lPLAs) [3–6].

Snake venom PLA_2_s of the Viperidae family, belong to the subgroup IIA, which share structural features with PLA_2_s present in inflammatory exudates in mammals [7, 8]. These snake venom PLA_2_s display a variety of pharmacological activities, such as myotoxic, neurotoxic, anticoagulant, hypotensive, hemolytic, platelet aggregation inhibiting, bactericidal, and proinflammatory activities [9–12]. Proteins of this subgroup can be further subdivided into two types: (i) catalytically active PLA_2_s, which have conserved residues at the catalytic network and at the calcium-binding loop, including Asp49, and (ii) catalytically inactive variants having a Lys instead of Asp at position 49 [10, 13, 14].

In addition, among the pro inflammatory activities, it was shown that various venom PLA_2_s are able to stimulate neutrophil chemotaxis, degranulate mast cells *in vitro*, induce prostaglandin production in peritoneal leukocytes, and activate macrophage functions [12, 15–19].

Macrophages play a central role in a wide variety of processes associated with tissue maintenance, antigen presentation, inflammation, and tissue repair [20]. Resident macrophages constitute one of the first lines of host defense and are present in many tissues. Upon stimulation, these quiescent cells are activated and display diverse cellular functions such as phagocytosis. This process consists of the uptake and destruction of invading microorganisms, an essential part of the host response against infections. It is initiated by engaging receptors on the surface of the macrophages which express various receptors that participate in target cell recognition and internalization of ligands [21]. There are four major classes of receptor-mediated phagocytosis: (a) Fc*γ* receptors, which recognize the Fc*γ* domain of IgG-coated particles; (b) complement receptor type 3 (CR3, also referred to as CD11b/CD18, Mac-1, and integrin *α*M*β*2) which recognizes complement-coated particles; (c) mannose receptors which recognize mannose and fucose on the surface of pathogens; and (d) *β*-glucan receptors or Dectin-1 which recognize *β*-glucans-bearing ligands [20, 21].

Subsequent to phagocytosis, an abrupt production of oxygen free radicals such as the superoxide anion occurs, referred to as an oxidative burst. The enzyme complex responsible for the production of this reactive oxygen species is the NADPH oxidase complex. When macrophages are stimulated, the membrane and cytosolic components of this complex associate on the phagosomal membranes to form the active oxidase complex [22–25]. The reactive oxygen species (ROS) formed, such as superoxide anion and hydrogen peroxide, are used by macrophages to kill ingested microorganisms [22–25]. Parallel to this reaction occurs lysossomal membrane destabilization involving increased fusion of lysosomes and phagosomes deriving from cellular stress. Lysossomal activities mediate several processes in cell feeding, homeostasis, and antimicrobial defense, which involve lysosome fusion with endosomes and phagosomes [26]. Furthermore, activated macrophages express a large amount of inclusions called lipid bodies (LBs). These LBs are neutral lipid storage organelles with key roles in cell signaling, intracellular trafficking, cell activation, and eicosanoid formation [27]. LBs were shown to characteristically increase both the size and number of cells associated with the inflammatory process. Additionally, macrophages, upon stimulation, release cytokines such as TNF-*α*, an important inflammatory mediator involved in many inflammatory reactions [28].

The present study was therefore designed to evaluate the effects of *Bothrops atrox* Lys-49 and Asp-49 PLA_2_s, acidic and basic proteins, on J774A.1 macrophage function. Our results indicate that these PLA_2_s, at noncytotoxic concentrations, are able to directly stimulate macrophages. Despite differences in enzymatic activity, the toxins induced inflammatory events and whether the enzyme is acidic or basic does not seem to contribute to these effects.

## 2. Materials and Methods

### 2.1. Chemicals and Reagents

Trypan blue, RPMI-1640, L-glutamine, gentamicin, zymosan, thiocarbohydrazide (TCH), nitroblue tetrazolium (NBT), mouse INF-*γ* and neutral red were purchased from Sigma (MO, USA). Fetal bovine serum was obtained from Cultilab (Brazil). Formaldehyde and OsO_4_^−^ (osmium tetroxide) were purchased from Electron Microscopy Sciences (PA, USA). DuoSet Elisa Mouse TNF-alpha was purchased from R&D Systems (Oxon, United Kingdom). All salts and reagents used were obtained from Merck (Darmstadt, Germany) with low endotoxin or endotoxin-free grades.

### 2.2. Venom

*Bothrops atrox* specimens collected around the city of Porto Velho, State of Rondônia, Brazil, were kept at the Fiocruz Rondônia bioterium in order to be used for venom production under authorization from IBAMA (license number 27131-1) and CGEN (license number 010627/2011-1). The crude venom was dehydrated and stored at a temperature of −20°C in the Amazon Venom Bank at CEBio.

### 2.3. Cell Line and Culture

The murine macrophage cell line J774A.1 was obtained from the Rio de Janeiro Cell Bank Collection (Brazil). Briefly, monolayers of J774A.1 macrophages were maintained in flasks in a RPMI-1640 medium supplemented with 100 *μ*g/mL gentamicin, 2 mM L-glutamine, and 10% heat-inactivated fetal bovine serum (FBS), in humidified air with 5% CO_2_ at 37°C.

### 2.4. Isolation and Biochemical Characterization of Phospholipases A_2_

Lys49 (BaTX-I) and Asp49 (BaTX-II) basic PLA_2_s as well as Asp49 (BaPLA_2_) acidic PLA_2_ from *Bothrops atrox* snake venom were purified according to the method previously described [29, 30]. *B. atrox* crude venom (100 mg) was dissolved in 1.0 mL of 0.05 M Tris-HCl buffer, pH 7.4, centrifuged at 755 ×g for 10 min at room temperature and the clear supernatant applied on a CM-Sepharose FF column (1 × 40 cm), which was previously equilibrated with the same buffer. Elution was carried out with a continuous gradient up to a concentration of 1.0 M of NaCl at a flow rate of 1.0 mL/min (HPLC, Akta Purifier, GE). Absorbance of the effluent solution was recorded at 280 nm. The last fraction with phospholipase and myotoxic activity was collected and freeze-dried. The acidic fraction (Peak 1) was freeze-dried and then diluted with 0.05 M Tris-HCl buffer, pH 7.4 containing 4 M NaCl prior to the next chromatographic step in a butyl-HP (Hitrap, 5 mL) column, previously equilibrated with the same buffer. The chromatography was carried out at a flow rate of 2.5 mL/min, with a decreasing gradient of NaCl (4 to 0 M), followed by Milli-Q H_2_O. The active fractions showing PLA_2_ activity were desalinized, lyophilized, and applied on a C18 reverse-phase column (Supelco, 250 mm × 4.6 mm), previously equilibrated with 0.1% TFA and then eluted with an acetonitrile-TFA gradient (0 to 70%), at a flow rate of 1.0 mL/min and monitored at *ƛ* = 280 nm.

Homogeneity was demonstrated using SDS-polyacrylamide gel electrophoresis under reducing conditions [31]. PLA_2_ activity of the proteins was evaluated *in vitro* by indirect erythrocyte lysis in agar containing human erythrocytes and egg yolk, as previously described [32]. A prior agreement from all participants involved was made in order to be included in the study, and the Center of Tropical Medicine Research (Rondonia, Brazil) Research Ethics Committees (number CAAE: 14204413.5.0000.0011) approved this study. The assay for myotoxicity was carried out using the creatine kinase (CK)-UV kinetic kit from Bioclin, Brazil [33].

### 2.5. Cytotoxic Assay

Cell viability was measured by trypan blue exclusion. In brief, J774A.1 macrophages were centrifuged at 200 ×g for 5 min at 4°C and the cell pellets were resuspended in 1 mL of RPMI-1640 medium supplemented with 100 *μ*g/mL gentamicin, 2 mM L-glutamine, and 10% fetal bovine serum (FBS). After counting, 2 × 10^5^ macrophages/80 *μ*L were added to plastic vials and incubated with 20 *μ*L of different concentrations of BaTX-I or BaTX-II or BaPLA_2_ (1.5; 3 and 6 *μ*g/mL) diluted in assay medium or RPMI (control), for 1 h and 4 h at 37°C in a humidified atmosphere (5% CO_2_). Then, 20 *μ*L 0.1% trypan blue was added to 100 *μ*L of macrophage suspension. The viable cell index was determined in a Neubauer's chamber by counting a total of 100 cells. Results were expressed as percentage of viable cells.

### 2.6. Adhesion Assay

Macrophage adhesion was assayed according to the procedure described by [34]. The cells (2 × 10^5^ cells/well) were cultured for 24 h in a supplemented medium. After incubation, the plates were washed three times with PBS and the adherent cells were fixed with methanol. After staining with 10% Giemsa solution for 10 min, the plates were washed with water, and the remaining dye was solubilized with methanol. Adherence of control cells was considered to be 100%. Absorbance was determined spectrophotometrically at 550 nm.

### 2.7. Detachment Assay

Macrophage detachment was assayed according to the procedure described by [34]. In this test, J774A.1 macrophages were plated in 96-well plates at a density of 2 × 10^5^ cells/well and allowed to attach for 24 h at 37°C in a 5% CO_2_ atmosphere. Nonadherent cells were removed by washing with PBS. After 24 h the cells were incubated with 200 *μ*L of supplemented RPMI (control) or BaTX-I or BaTX-II or BaPLA_2_ (6 *μ*g/mL) diluted in RPMI at 37°C in a humidified atmosphere (5% CO_2_) for 1 h. After another washing with PBS, the cells were stained with 50 *μ*L of 0.1% Giemsa for 40 min. Finally, the cells were washed with PBS and 100 *μ*L of 100% methanol was added to solubilize the dye. Adherence of the remaining control cells was considered to be 100%. Absorbance was read at 550 nm. The results were expressed as absorbance.

### 2.8. Phagocytic Activity of J774A.1 Macrophages

This test was performed according to [12]. In brief, J774A.1 macrophages were plated on 13 mm diameter glass coverslips (Glass Tecnica, Brazil) in 24-well plates at a density of 2 × 10^5^ cells per coverslip and allowed to attach for 2 h at 37°C under a 5% CO_2_ atmosphere. Nonadherent cells were removed by washing with PBS. Cell monolayers were cultured for 1 h with RPMI-1640 supplemented with 100 *μ*g/mL gentamicin, 2 mM L-glutamine, and 10% FBS at 37°C and 5% CO_2_ and then incubated with RPMI (control) or different concentrations of BaTX-I or II or BaPLA_2_ (6 *μ*g/mL) diluted in RPMI. After washing in cold PBS the monolayers were incubated for 40 min at 37°C and 5% CO_2_ with serum-opsonized zymosan, prepared as described below, and unbound particles were removed by washing with PBS. Cells were fixed with 2.5% glutaraldehyde for 15 min at room temperature and the coverslips were mounted on microscope slides. The extent of phagocytosis was quantified by contrast phase microscopic observation. At least 200 macrophages were counted in each determination and those containing three or more internalized particles were considered positive for phagocytosis. Results were presented as the percentage of cells positive for phagocytosis.

To prepare serum-opsonized zymosan, zymosan particles, obtained from yeast cell walls, were suspended in PBS at a concentration of particles of 5.7 mg/mL. For opsonization, 2 mL of zymosan particles was mixed with 2 mL normal mouse serum and incubated for 30 min at 37°C. The serum-opsonized zymosan particles were then centrifuged at 200 g for 10 min and suspended in PBS in preparation for the phagocytosis assay. Mouse serum from swiss mice used in the present study was approved by the Tropical Pathology Research Institute Ethics Committee on Animal Use (number 08/2008).

### 2.9. Staining and Counting of Lipid Bodies

Macrophages were plated on 13 mm diameter glass coverslips (Glass Tecnica, Brazil) in 24-well plates at a density of 2 × 10^5^ cells per coverslip and allowed to attach for 2 h at 37°C in a 5% CO_2_  atmosphere. Nonadherent cells were removed by washing with PBS. Cell monolayers were incubated with supplemented RPMI (control) or BaTX-I or BaTX-II or BaPLA_2_ (6 *μ*g/mL) diluted in RPMI for 60 min at 37°C and 5% CO_2_. In a set of experiments, J774A.1 macrophages were submitted to phagocytosis according to item 2.8 and then the lipid bodies were stained. After washing in cold PBS the monolayers were fixed with 3.7% formaldehyde in PBS, pH 7.4, for 15 min at room temperature, rinsed in 0.1 M cacodylate buffer, pH 7.4, stained in 1.5% OsO_4_ (30 min), rinsed in dH_2_O, immersed in 1.0% thiocarbohydrazide (5 min), rinsed in 0.1 M cacodylate buffer, restained in 1.5% OsO_4_ (3 min), rinsed in dH_2_O, and then dried and mounted on microscope slides. The morphology of fixed cells was observed, and lipid bodies were counted using phase contrast microscopy with an objective lens at a magnification of 100 in 50 consecutively scanned macrophages. The results were expressed as the mean number of lipid bodies per cell.

### 2.10. Production of Superoxide Anion by J774A.1 on Glass Coverslips

In this test, J774A.1 the concentration of macrophages was adjusted to 2 × 10^5^/100 *μ*L and they were incubated with 100 *μ*L RPMI containing 0.1% NBT (control) or 100 *μ*L of different concentrations of BaTX-I or BaTX-II or BaPLA_2_ (6 *μ*g/mL), diluted in RPMI containing 0.1% NBT, and incubated for 1 h at 37°C and 5% CO_2_. At the end of the incubation period, the cells were centrifugated in a cytospin for 5 min at 180 ×g and then fixed with methanol for 5 min. Finally, the cells were stained with 0.1% safranin for 5 min. At least 100 macrophages were counted and those containing blue formazan crystals were considered positive. The results were expressed in a percentage.

### 2.11. Lysosomal Volume

This test, which evaluates the vesicles of the endocytic compartment by lysosomal retention of neutral red, was performed according to [35]. J774A.1 macrophages were incubated for 15, 30, and 60 minutes in the presence of RPMI (control) or BaTX-I or BaTX-II or BaPLA_2_ (6 *μ*g/mL) at 37°C in a humidified atmosphere (5% CO_2_). Then, the cells were washed by centrifugation and incubated with 0.04% neutral red (stock solution 2% = 20 mg dissolved in 1 mL DMSO). After this the macrophages were washed again by centrifugation and the dye was solubilized by adding 200 *μ*L of extraction solution (acetic acid 10% and 40% ethanol in distilled water). The cells were incubated for 30 minutes and then the samples were read on a spectrophotometer at 550 nm. The results were expressed in percentage, considering the control group to have 100% absorbance.

### 2.12. Tumor Necrosis Factor-*α* (TNF-*α*) Quantification

J774A.1 macrophages (2 × 10^5^/50 *μ*L) were incubated with BaTX-I or BaTX-II or BaPLA_2_ (6 *μ*g/mL) or RPMI (control) or mouse INF-*γ* (1 *μ*g/mL, positive control) for 4 hours at 37°C in a humidified atmosphere (5% CO_2_). After centrifugation the supernatant was used to determine TNF-*α* levels by specific EIA, as described by [36]. Briefly, 96-well plates were coated with 100 *μ*L of the first capture monoclonal antibody antitumor necrosis factor-*α* (4 *μ*g/mL) and incubated for 18 hours at 37°C. Following this period, the plate was washed with washer buffer (PBS/Tween20). After that, 200 *μ*L of blocking buffer, containing 5% bovine serum albumin (BSA) in PBS/Tween20, was added to the wells and the plates were incubated for 1 hour at 37°C. Following this period, the wells were washed and 50 *μ*L of either samples or standard was dispensed into each well and the plates incubated for 2 hours at 37°C. After this period, the plate was washed and 100 *μ*L of detection antibody TNF-*α* (250 ng/mL) was added for 2 hours at 37°C. After incubation and washing, 100 *μ*L of streptavidin-peroxidase was added, followed by incubation and addition of the substrate (100 *μ*L/mL 3,3′,5,5′-tetramethylbenzidine). Finally, sulfuric acid (50 *μ*L) was added to stop the reaction. Absorbances at 540 and 450 nm were recorded and concentrations of TNF-*α* were estimated from standard curves prepared with recombinant TNF-*α*. The results were expressed as pg/mL of this cytokine.

### 2.13. Statistical Analyses

Means and S.E.M. of all data were obtained and compared using two way ANOVA, followed by a Tukey test with significance probability levels of less than 0.05.

## 3. Results

### 3.1. Isolation and Biochemical Characterization of Myotoxins

**Figure 1:**
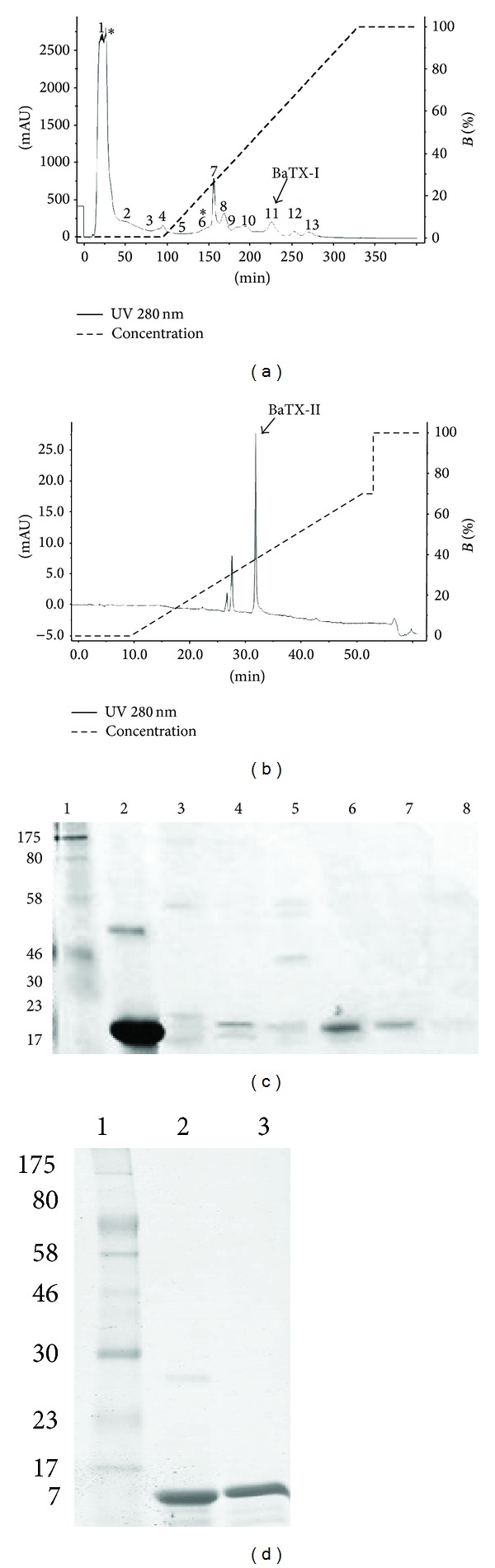
(a) Chromatographic profile of *Bothrops atrox* venom. The crude venom of *B*. *atrox* after being solubilized was applied on CM-Sepharose (400 mm × 10 mm) previously equilibrated with Tris 50 mM pH 7.4 and eluted under a gradient of NaCl (0–1 M) in the same buffer, in 5 column volumes. The fractions with PLA_2_ activity are identified with (∗). (b) High performance chromatographic profile. Fraction 6 obtained from CM-Sepharose was solubilized in 0.1% TFA (solvent A) and applied on a C18 column (discovery 25 mm × 46 mm, 5 *μ*, 300 Å) previously equilibrated with the same buffer and eluted with 0.1% TFA in 99.9% acetonitrile (solvent B) under a gradient 0–70% and flow of 1 mL/min. Both chromatograms were monitored with absorbance at 280 nm. (c) SDS-PAGE 12. 5%: samples: 1 molecular weight marker; 2 BthTX-I; 3 fraction 8; 4 fraction 9; 5 fraction 10; 6 11 fraction (BaTX-I); 7 fraction 12. (d) SDS-PAGE 12.5% in reducing conditions: electrophoretic analysis of basic Asp49 PLA_2_ from *B*. *atrox*. Samples: 1 molecular weight; 2 BthTX-I; 3 BaTX-II.

**Figure 2:**
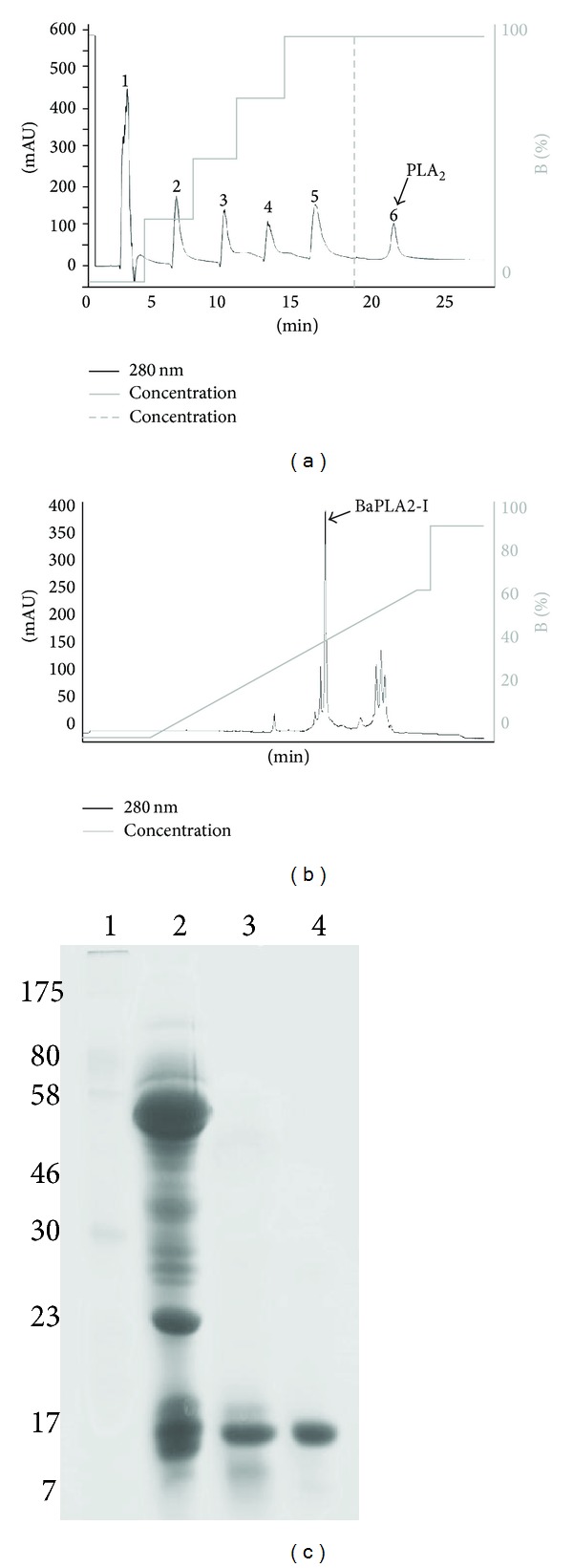
(a) Chromatography profile on hydrophobic interaction resin (Butyl-Sepharose HP 50 mm × 10 mm). Fractions 1, 2, and 3 obtained from CM-Sepharose chromatography were pooled and solubilized in 20 mM Ambic plus 4 M NaCl (buffer A) and applied on a Butyl-Sepharose HP column previously equilibrated with the same buffer and eluted with 20 mM Ambic (buffer B) using a step gradient of 0, 25, 50, 75, and 100%. Subsequently, a step using Milli-Q water was performed. (b) High performance chromatographic profile. Fraction 6 obtained from a Butyl-Sepharose HP column was solubilized in 0.1% TFA (solvent A) and applied on a C18 column (discovery 25 mm × 46 mm, 5 *μ*, 300 Å) previously equilibrated with the same buffer and eluted with 0.1% TFA in 99.9% acetonitrile (solvent B) under a gradient of 0–70% and flow of 1 mL/min. Both elutions were monitored with absorbance at 280 nm.

*Bothrops atrox* crude venom (100 mg) was applied on a CM-Sepharose ion-exchange column, previously equilibrated with 0.05 M Tris-HCl buffer, pH 7.4, and then eluted with a continuous gradient up to a concentration of 1.0 M of NaCl. Fraction CM-11, with myotoxic activity, was named BaTX-I (Figures 1(a) and 1(c)). The fractions CM-6 and CM-1, with phospholipase A_2_ activity, were further fractionated on reverse phase C18 (Figure 1(b)) and Butyl-Sepharose (Figure 2(a)) columns, respectively. The fraction CM-6-3 (Figure 1(b)), with PLA_2_ and myotoxic activities, was named BaTX-II (Figure 1(d)). Fraction CM-1-6 was applied on a RP-HPLC C18 column and resolved into one main peak with only PLA_2_ activity, named BaPLA_2_ (Figures 2(b) and 2(c)). The homogeneity of these proteins was further demonstrated by SDS-PAGE. The purified myotoxins consisted of a single polypeptide chain with an apparent approximate molecular weight of 14,500 Da.

### 3.2. Effect of BaTX-I, BaTX-II, and BaPLA_2_ on Macrophage Viability

**Figure 3:**
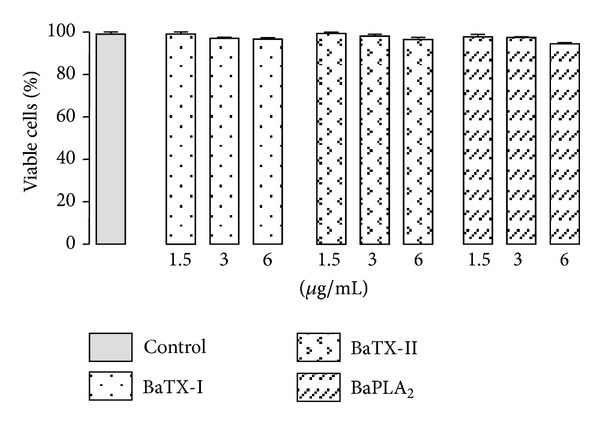
Effect of BaTX-I, BaTX-II, and BaPLA_2_ on macrophage viability. 2 × 10^5^ J774A.1 macrophages were incubated with different concentrations of toxins or RPMI (control) for 60 minutes at 37°C in a humidified atmosphere of 5% CO_2_. The viability of macrophages was assessed by a Trypan blue exclusion test. Values represent the mean ± S.E.M. from 3 independent experiments.

To test the toxicityof PLA_2_s on J774A.1 macrophages, the effect of 1 and 4 hours of incubation of several concentrations of BaTX-I or BaTX-II or BaPLA_2_ was investigated. As shown in Figure 3, the incubation of secretory PLA_2_s at concentrations of 1.5, 3, and 6 *μ*g/mL for 1 h and 4 h (data not shown) did not affect J774A.1 macrophage viability.

### 3.3. Effect of BaTX-I, BaTX-II, and BaPLA_2_ on Macrophage Adhesion

**Figure 4:**
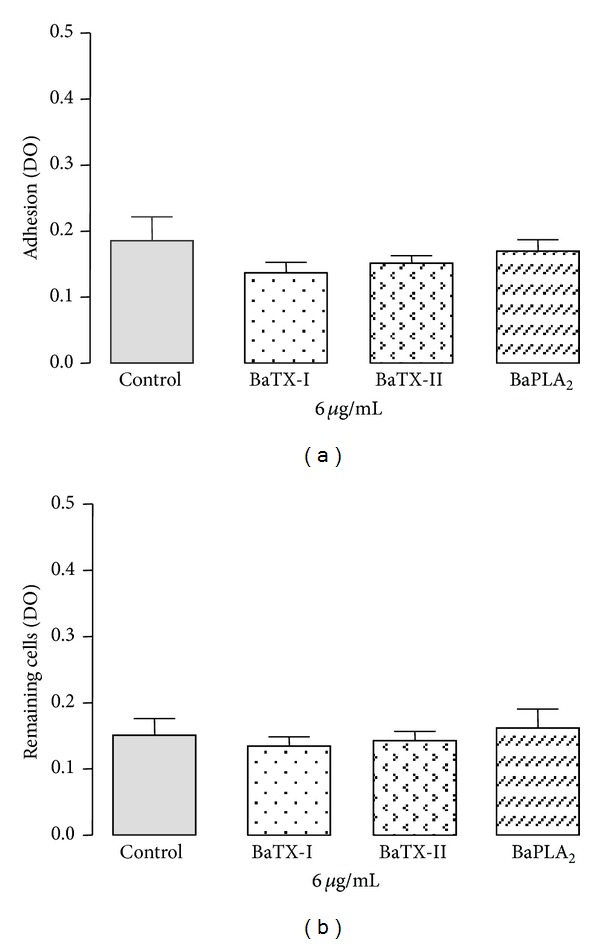
Effect of BaTX-I, BaTX-II, and BaPLA_2_ on J774A.1 macrophage adhesion and detachment. For the adhesion test (a), 2 × 10^5^ macrophages were incubated for 60 minutes with toxins (6 *μ*g/mL) or RPMI (control) at 37°C in a humidified atmosphere of 5% CO_2_. For the detachment test (b), macrophages were incubated with RPMI for only 24 hours and then for 60 minutes with toxins (6 *μ*g/mL) or RPMI (control) at 37°C in a humidified atmosphere of 5% CO_2_. Adhered cell levels were determined by optical density (550 nm) being proportional to the amount of incorporated Giemsa dye. Values represent the mean ± S.E.M. from 3 independent experiments.

To investigate the effect of BaTX-I, BaTX-II, and BaPLA_2_ on macrophage adhesion, the cells were incubated with 6 *μ*g/mL of either PLA_2_s (a noncytotoxic concentration) or RPMI (control). As shown in Figure 4(a), the toxins did not affect the adhesion of the J774A.1 macrophages.

### 3.4. Effect of BaTX-I, BaTX-II, and BaPLA_2_ on Macrophage Detachment

To test whether these secretory PLA_2_s of *Bothrops atrox* snake venom affect the ability of J774A.1 macrophages to detach, the cells were incubated with BaTX-I or BaTX-II or BaPLA_2_ at a noncytotoxic concentration (6 *μ*g/mL) or RPMI (control). As shown in Figure 4(b), the studied PLA_2_s did not affect the ability of the macrophages to detach.

### 3.5. Effect of BaTX-I, BaTX-II, and BaPLA_2_ on Phagocytosis by Macrophages

**Figure 5:**
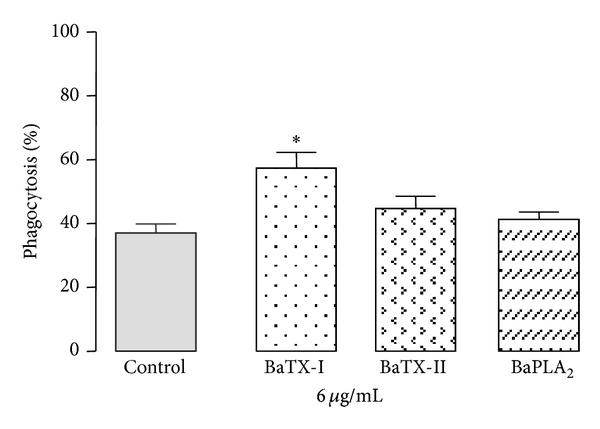
Effect of BaTX-I, BaTX-II, and BaPLA_2_ on phagocytosis of opsonized zymosan by J774A.1 macrophages. 2 × 10^5^ macrophages were incubated for 60 minutes with toxins (6 µg/mL) or RPMI (control) at 37°C in a humidified atmosphere of 5% CO_2_ before addition of opsonized zymosan particles. Values represent the mean ± S.E.M. from 3 independent experiments *P ≤ 0.05 compared with control (ANOVA).

In order to assess the ability of BaTX-I, BaTX-II, and BaPLA_2_ to stimulate complement receptor-mediated phagocytosis, the uptake of serum-opsonized zymosan particles was determined in adherent J774A.1 macrophages treated with noncytotoxic concentration of toxins or with RPMI (control). As shown in Figure 5, macrophages incubated with RPMI showed an average phagocytosis of serum-opsonized zymosan particles of 37%. Incubation of macrophages with BaTX-I induced a significant increase in J774A.1 macrophage phagocytosis of serum-opsonized zymosan at a concentration of 6 *μ*g/mL. On the other hand, incubation of macrophages with BaTX-II and BaPLA_2_, at a concentration of 6 *μ*g/mL, resulted in 44.6 ± 3.8% and 41.3 ± 2.3%, of phagocytosis of serum-opsonized zymosan, respectively. These values were not significant compared to control.

### 3.6. Effect of BaTX-I, BaTX-II, and BaPLA_2_ on Superoxide Anion (O_2_^−^) Production by Macrophages

**Figure 6:**
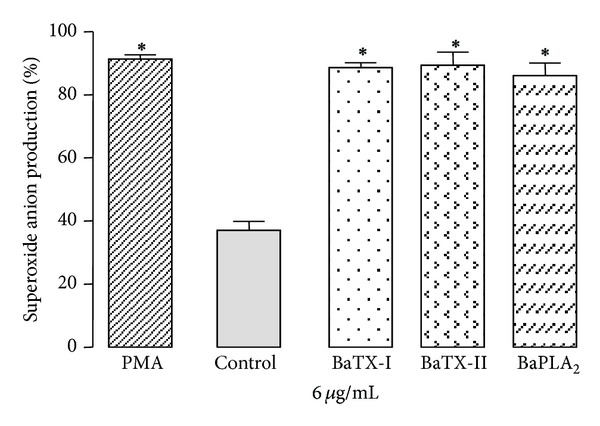
Effect of BaTX-I, BaTX-II, and BaPLA_2_ on superoxide production by J774A.1 macrophages. 2 × 10^5^ macrophages were incubated with RPMI, 0.1% NBT, 10 uL of PMA (500 ng/mL) (positive control) or toxins (6 µg/mL) for 60 minutes at 37°C in a humidified atmosphere of 5% CO_2_ for the formation of formazan crystals resulting from the reduction of NBT by superoxide. The crystals were solubilized and the absorbance of the supernatant was determined at 620 nm in a spectrophotometer. Data were expressed as O.D. Values represent the mean ± S.E.M. from 3 independent experiments. *P ≤ 0.05 compared with control (ANOVA).

To investigate the ability of BaTX-I, BaTX-II, and BaPLA_2_ to induce the production of the superoxide anion by J774A.1 macrophages, the cells were incubated with a noncytotoxic concentration of BaTX-I, BaTX-II, or BaPLA_2_, or with RPMI (control), in the presence of NBT. As shown in Figure 6, J774A.1 macrophages incubated with RPMI showed an average superoxide anion production of 37 ± 2.8%. Incubation of macrophages with BaTX-I, BaTX-II, and BaPLA_2_ at a concentration of 6 *μ*g/mL, induced significant production of O_2_^−^ by J774A.1 macrophages.

### 3.7. Effect of BaTX-I, BaTX-II, and BaPLA_2_ on Macrophage Lipid Bodies

**Figure 7:**
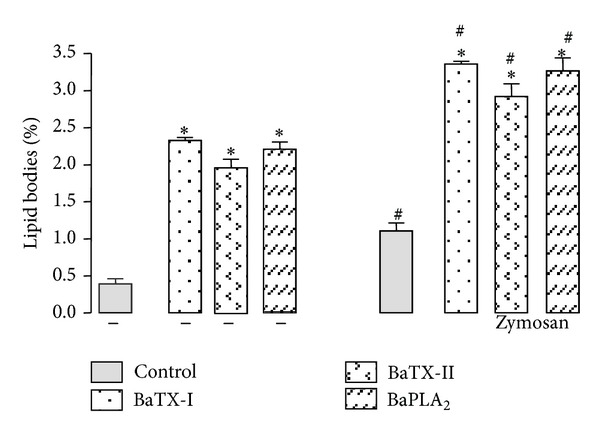
Effect of BaTX-I, BaTX-II, and BaPLA_2_ on lipid body formation by J774A.1 macrophages. 2 × 10^5^ macrophages were incubated with toxins (6 µg/mL) or RPMI (control) for 60 minutes at 37°C in a humidified atmosphere of 5% CO_2_ before the addition of opsonized zymosan particles. The number of lipid bodies stained with osmium tetroxide within macrophages was determined using phase-contrast microscopy. Values represent the mean ± S.E.M. from 3 independent experiments. *P ≤ 0.05 compared with control and ^#^ *P* ≤ 0.05 compared with control submitted to phagocytosis (ANOVA).

To determine whether these enzymes induce the formation of lipid bodies in J774A.1 macrophages, the cells were incubated with noncytotoxic BaTX-I, BaTX-II, or BaPLA_2_, or with RPMI (control) for 1 h. After that, the cells were submitted to phagocytosis and lipid bodies were counted. As shown in Figure 7, the incubation of macrophages with the toxins increased the number of lipid bodies compared to the control. In the cells submitted to phagocytosis, the amount of lipid bodies was significantly higher than cells not submitted to phagocytosis.

### 3.8. Effect of BaTX-I, BaTX-II, and BaPLA_2_ on Macrophage Lysossomal Volume

**Figure 8:**
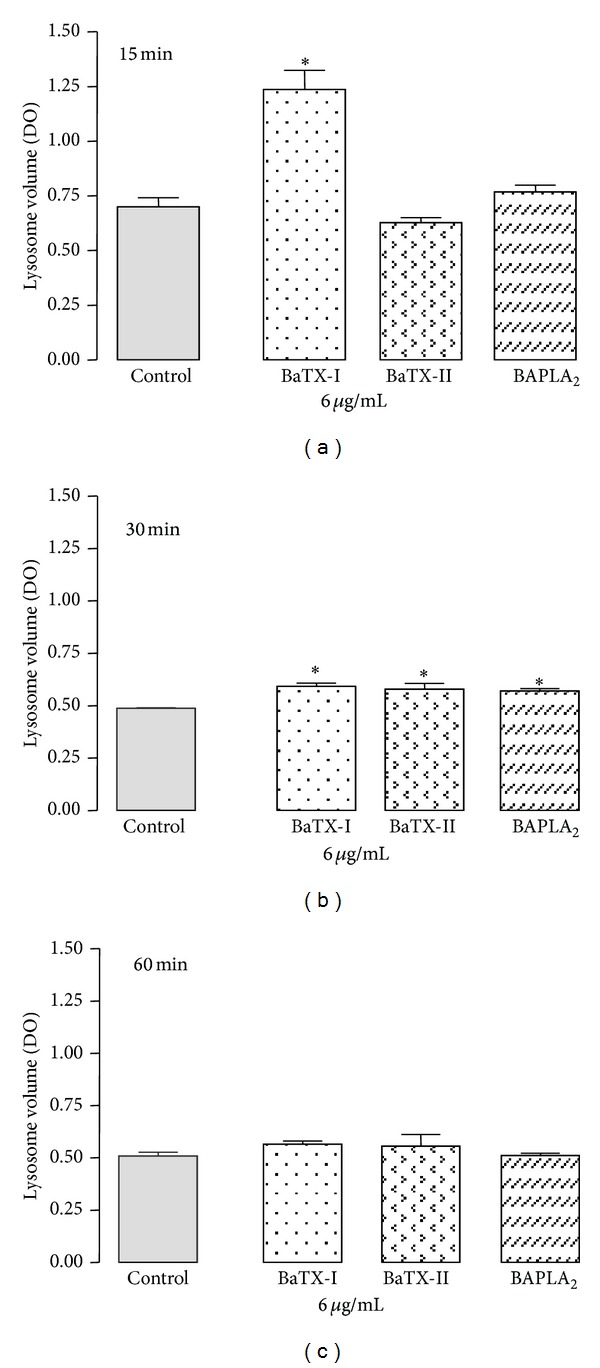
Effect of BaTX-I, BaTX-II, and BaPLA_2_ on J774A.1 macrophage lysosomal volume. 2 × 10^5^ macrophages were incubated with the toxins (6 µg/mL) or RPMI (control) for 15, 30, and 60 minutes at 37°C in a humidified atmosphere of 5% CO_2_. After that, the macrophages were incubated with 0.04% neutral red. The incorporated dye was solubilized and the absorbance of the supernatant was determined at 505 nm in a spectrophotometer. Values represent the mean ± S.E.M. from 3 independent experiments. *P ≤ 0.05 compared with control (ANOVA).

To assess the effect of these secretory PLA_2_s on macrophage lysossomal volume, the cells were stimulated with noncytotoxic concentrations of BaTX-I, BaTX-II, or BaPLA_2_, or with RPMI (control) for 15, 30, and 60 min. As shown in Figure 8(a), incubation with the toxin BaTX-I at a concentration of 6 *μ*g/mL for 15 min increased the lysossomal volume in J774A.1 macrophages compared to the control. The toxins BaTX-II and BaPLA_2_ did not affect macrophage lysossomal volume at this time. However, after incubation for 30 min, the three toxins induced a significant increase in macrophage lysossomal volume, as shown in Figure 8(b). Incubation of the cells with secretory PLA_2_s at a concentration of 6 *μ*g/mL for 1 h did not affect the lysossomal volume in macrophages, as shown in Figure 8(c).

### 3.9. Effect of BaTX-I, BaTX-II, and BaPLA_2_ on TNF-*α* Production by Macrophages 

**Figure 9:**
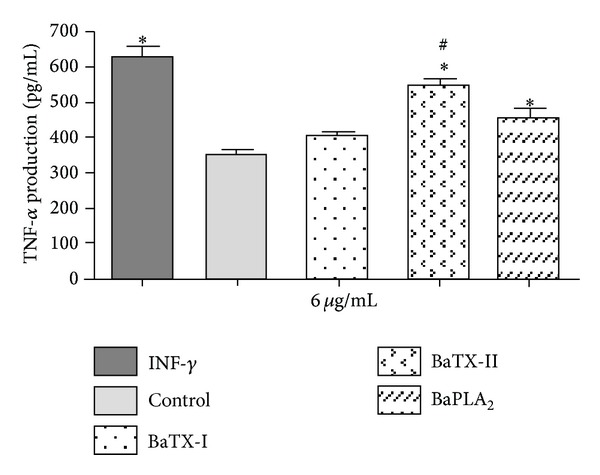
Effect of BaTX-I, BaTX-II, and BaPLA_2_ on J774A.1 macrophage TNF-*α* production. 2 × 10^5^ macrophages were incubated with RPMI (control) or INF-*γ* (1 µg/mL) (positive control) or toxins (6 µg/mL) for 4 hours. The concentrations of TNF-*α* in the supernatant were quantified by specific EIA. The results were expressed as pg/mL of TNF-*α* and represent the mean ± S.E.M from 3 independent experiments. *P ≤ 0.05 compared to control and ^#^ *P* ≤ 0.05 compared to BaTX-II (ANOVA).

To investigate the effect of these secretory PLA_2_s on TNF-*α* production by macrophages, the cells were stimulated with IFN-*γ* (1 *μ*g/mL, positive control) or noncytotoxic concentrations of BaTX-I or BaTX-II or BaPLA_2_ or RPMI (control) for a period of 4 hours. Soon after, the supernatant was collected and used to determine TNF-*α* concentration using an enzyme immunoassay. As shown in Figure 9, the incubation with the enzymatically active toxins BaTX-II and BaPLA_2_ induced an increase in TNF-*α* production by macrophages, although BaTX-II was more effective than BaPLA_2_ in maintaining this effect. The enzymatically inactive toxin BaTX-I did not induce any significant increase in the production of TNF-*α* in the studied period.

## 4. Discussion

Both acidic and basic PLA_2_s can be found in snake venoms in variable proportions depending on the snake species. The basic PLA_2_ isoforms appear to have the highest toxicity and the acidic PLA_2_ isoform usually has higher catalytic activity. To date, all acidic PLA_2_s purified from Viperidae venoms present an Asp residue at position 49 [37, 38]. In this study, we purified two basic myotoxic PLA_2_s from *B. atrox *venom, named BaTX-I (Lys-49), and BaTX-II (Asp-49) and an acidic PLA_2_ named BaPLA_2_ (Asp-49), by one-, two-, or three-step chromatography. The quality of the final sample is crucial for further studies involving structure and function. Several PLA_2_s from *Bothrops *venoms have been exhaustively purified using a combination of chromatographic methods: gel filtration, ion exchange, RP-HPLC, and affinity chromatography [10, 39].

Based on this, we performed a study in order to evaluate the effect of three sPLA_2_s BaTX-I (Lys-49), BaTX-II (Asp-49), and BaPLA_2_ (Asp-49) on the functionality of the murine macrophage cell line J774A.1.

For this, we assessed, first, the cytotoxic activity of these phospholipases. The viability assay using the trypan blue exclusion method demonstrated that toxins at concentrations of 1.5, 3, and 6 *μ*g/mL showed no cytotoxic effect on macrophages during 1 hour of incubation. Thus, for the realization of the other proposed experiments, the highest concentration, 6 *μ*g/mL, was chosen. It is noteworthy that the same concentration was used in the study of macrophage activation by Zuliani et al. [12] and Setúbal et al. [19]. The three PLA_2_s used had the same effect on macrophages. Still, it is worth noting that the acidic or basic character of these toxins did not influence the results.

Corroborating this data, Lomonte and Gutierrez [40] showed that two basic PLA_2_s isolated from the venom of *Bothrops asper* (MT-II and MT-III) exert cytotoxic effects only at high concentrations. Also, in the study by Zuliani et al. [12] it was shown that thioglycollate elicited murine peritoneal macrophages incubated with concentrations less than 25 *μ*g/mL of MT-II and MT-III, for a period of one hour, did not show compromised viability. The study also showed that the enzymatically active toxin MT-III (Asp-49) is more toxic than the enzymatically inactive toxin MT-II (Lys-49). In addition, Setúbal et al. [19] examined the effects of BaltTX-I and BaltTX-II on thioglycollate elicited murine peritoneal macrophage viability. These two basic PLA_2_ did not affect macrophage viability, indicating their low toxicity on this cell type.

Macrophages, a professional phagocyte, play an important role in the innate immune response against invading pathogens in the resolution of inflammation and maintenance of tissue homeostasis. This cell type has a central role in the inflammatory reaction, producing microbicidal agents and exerting its main function: phagocytosis. For this to occur efficiently, macrophages need to adhere to a substrate and then modify their cell morphology, gaining a more flattened shape. This step is very important and allows the macrophage to spread and increase its contact surface area with the particle to be phagocytosed. Thus, our study evaluated the effect of the three phospholipases on the ability of macrophages to adhere and to detach. The results showed that BaTX-I, BaTX-II, and BaPLA_2_ did not affect the ability of macrophages to adhere and to detach from support in the period studied.

The literature reports that PLA_2_s have an important role in cell adhesion. The use of inhibitors of PLA_2_s such as p-bromophenacyl bromide reduced monocyte adhesion and affected the expression of Mac-1 in neutrophils [41, 42] as well as the spreading of these cells [43]. On the other hand, two nontoxic enzymes with high PLA_2_ activity isolated from *Cerastes cerastes* snake venomcalled CC-PLA_2_-I and CC-PLA_2_-II inhibited the migration and adhesion of IGR39 melanoma and HT1080 fibrosarcoma cells to fibrinogen and fibronectin [44]. Bazaa et al. [45] demonstrated the action of another PLA_2_, called MVL-PLA_2_, isolated from *Macrovipera lebetina* Transmediterranea. In this study, the enzyme was also able to inhibit the adhesion and migration of tumor cells to fibrinogen and fibronectin, but not to collagen type I. These effects were mediated by the specific inhibition of *α*5*β*1, *α*v*β*6, and *α*v*β*3 integrins. These data show that integrins may be a specific target for phospholipases' mechanism of action in tumor cell lines. Despite the ability of detachment, our data showed that cells remained viable after incubation with toxins remaining adhered to the plate surface. It is important for the process of phagocytosis to occur effectively. Then, macrophages need to adhere to a substrate to subsequently modify their cellular morphology from a round shape into a flattened shape [46].

Phagocytosis is an extremely complex process and may be mediated by several receptors such as the receptor for the Fc portion of IgG (Fc*γ*Rs), the mannose receptor (MR), the *β*-glucan receptor (Dectin-1), and complement receptor type 3 (CR3). Accordingly, we evaluated the effect of PLA_2_s on phagocytosis via the complement receptor, using opsonized zymosan particles. Our results showed that only BaTX-I, a Lys-49 toxin, induced a significant increase in phagocytosis via the complement receptor. The others toxins, BaTX-II and BaPLA_2_, both Asp-49, did not affect this process. The acidic or basic character does not seem to affect this parameter. These results are in agreement with those obtained by Zuliani et al. [12]. The authors showed that MT-II, a Lys-49 isolated from the venom of *Bothrops asper*, also considerably increases phagocytosis via the complement receptor, while MT-III, an Asp-49, only stimulates phagocytosis via mannose receptors. Similar results were obtained by Setúbal et al. [19]. These authors showed that BaltTX-I, a Lys49, but not BaltTX-II, an Asp-49 enzyme, is able to directly stimulate phagocytosis via the complement receptor. Thus, it is important to note that enzymatic activity does not seem to be essential for increasing the phagocytic capacity via CR3, since only catalytically inactive phospholipases were able to affect this parameter. The mechanism by which Lys49 PLA_2_ homologs activate the process of phagocytosis has not been clarified. There are two hypotheses for the action of these enzymatically inactive toxins. One is the interaction of the enzyme with the receptor, leading to its internalization. The other hypothesis is that the PLA_2_s have the ability to induce perturbations in the cell membrane, which activate signaling pathways culminating in an increase of phagocytosis. Thus, further studies are needed to elucidate the mechanism of action of these enzymes, which makes them extremely interesting from a scientific standpoint.

Concomitantly with phagocytosis, there is an increase in the oxidative metabolism of macrophages, called a respiratory burst that is mediated by the NADPH oxidase enzyme complex. The high O_2_ consumption associated with rapid activation of NADPH oxidase triggers the production of reactive oxygen species (ROS) such as the superoxide anion (O_2_^−^) and hydrogen peroxide (H_2_O_2_), which are potent microbicidal agents [47]. Therefore, this study evaluated the PLA_2_ action on superoxide anion production by J774A.1 macrophages. The results showed that all three toxins significantly increased superoxide anion release compared with the unstimulated control. The increase was similar to that observed in cells incubated with PMA, a positive control. There was no difference between the Asp49 and Lys49 phospholipases A_2_, suggesting that catalytic activity may not be essential to superoxide anion production. Once again, it is clear that the enzyme's acidic or basic character does not appear to contribute to this effect.

According to Setúbal et al. [19] the excessive release of superoxide can be harmful to tissues involved in inflammation. In this study the authors showed that BaltTX-I and BaltTX-II, isolated from *Bothrops alternatus* venom, induced a significant release of superoxide anion compared with the negative control (RPMI medium), but there was no difference when compared to PMA (a positive control), indicating that the myotoxins are able to activate the respiratory burst. Moreover, Zuliani et al. [12] showed that MT-II and MT-III, isolated from *B*. *asper* venom, induce the release of H_2_O_2_, another reactive oxygen intermediate produced from the superoxide anion. In this case, MT-III's activity was more pronounced than that of MT-II. This increase in microbicidal agent production induced by PLA_2_s may be considered one of the key points for the development of the intense local inflammatory process observed in *Bothrops* envenoming.

It is possible that the increase in phagocytic index is also associated with an increase in the endocytic compartment, contributing to an increase in the microbicidal activity of the macrophages, since destabilization of lysosomal membranes involves an increase in lysosome and phagolysosome fusion, often associated with stress derived from the action of pro-oxidant agents and pathological conditions. Accordingly, experiments were performed to assess whether the toxins are able to induce an increase in lysosomal volume. The macrophages were incubated with toxins for 15, 30, and 60 minutes. In evaluating the time course of the effect of the three toxins, it can be seen that increasing the endocytic compartment of macrophages occurs quite sharply in the first 15 minutes after stimulation and that enzymatic activity is not essential for this effect, since only BaTX-I, which is enzymatically inactive, changed this parameter. The enzymatically active phospholipases BaTX-II and BaPLA_2_ were only able to induce an increase in lysosomal volume after 30 minutes of incubation. During this period, BaTX-I was also effective in maintaining this effect. After being stimulated for 1 hour with phospholipases, macrophages showed no change in their endocytic compartment. This may suggest that, after a period of 30 minutes, the PLA_2_ action decreases and the volume of the cells' lysosomal compartments returns to their baseline level. Despite the fact that the lipolytic activity of PLA_2_ often affects several membrane structures including lysosomes, in our studies, enzymatic activity does not appear to interfere with the increase in lysosomal volume, nor does the acidic or basic character of these enzymes. Studies by Burlando et al. [26] showed that a calcium-independent cPLA_2_ is one of the agents responsible for the lysosomal membrane destabilization induced by estradiol. The destabilization of the membrane leads to the fusion of the lysosome with other organelles such as the endosome. This process, as evidenced by Mayorga et al. [48], involves the action of a PLA_2_. Thus, the data suggest that these enzymes can induce changes in the vacuole-lysosomal system.

The literature shows that, in the presence of inflammation, activated leukocytes induced an increase of organelles called lipid bodies. These organelles compartmentalize different groups of proteins involved in lipid metabolism and in cell signaling, in addition to containing the complex enzyme COX and 5-LO, and even AA, cPLA_2_, MAPK, and PI3K [49, 50]. Thus, the actions of BaTX-I, BaTX-II, and BaPLA_2_ on lipid body formation in J774A.1 macrophages were evaluated. The data obtained show that all three phospholipases were able to increase the number of lipid bodies present in the macrophages. This action occurred independently of enzymatic activity and no statistical difference was observed between the toxins. Similar results were obtained by Leiguez et al. [51] and Giannotti et al. [52]. These authors showed that MT-III, an Asp49, and MT-II, a Lys49, both isolated from *B. asper* venom were able to induce a marked increase in lipid bodies formation in macrophages. Therefore, we evaluated the action of these PLA_2_s in macrophages undergoing phagocytosis. The results showed that the number of lipid bodies inside macrophages that phagocytosed was greater than that found in macrophages which were not subjected to the process. The BaTX-I was slightly more efficient than the others in the maintenance of this effect. In relation to enzymatic activity, the three phospholipases were able to induce these events regardless of catalytic ability. Again, there was no difference in the acidic or basic character of these enzymes. The data suggest that the activation of complement receptors during phagocytosis stimulates lipid body formation in macrophages, also induced by the action of sPLA_2_s. Thus, PLA_2_s, in addition to releasing the AA of membrane phospholipids, can hydrolyze phosphatidylcholine and cause lyso-PAF release, which is the precursor of platelet activating factor (PAF). Data from the literature indicate that this inflammatory mediator, with platelet aggregating activity, is involved in signaling pathways that favor the formation of lipid bodies and the release of mediators such as LTC_4_ and PGE_2_ [50]. Additional studies to determine the role of lipid bodies in the process of phagocytosis and the mechanisms of formation of these organelles are required. These details also have significance for the functionality of the leucocytes during inflammatory reactions.

Besides the increase in the number of lipid bodies, activated macrophages show an increased production of cytokines with important roles in inflammatory responses. One of them is TNF-*α*, a potent inflammatory mediator capable of inducing proliferation of fibroblasts producing other cytokines, activating leukocytes, and stimulating the generation of reactive oxygen species. Thus, we evaluated the action of the three phospholipases, BaTX-I, BaTX-II, and BaPLA_2_, on TNF-*α* production by J774A.1 macrophages. The data showed that BaTX-II and BaPLA_2_ induced a significant increase in TNF-*α* production by macrophages after 4 hours of incubation, and BaTX-II was somewhat more effective than BaPLA_2_ in inducing the production of this cytokine. Both toxins are enzymatically active Asp49 homologs, whereas the first is basic and the latter is acidic. The results also show that BaTX-I, an enzymatically inactive Lys49 PLA_2_ homolog, did not induce any increase in the production of this cytokine in the studied period. These data are in agreement with Zuliani et al. [11], in which MT-III, an Asp49, isolated from *B*. *asper*, induced an increase in the production of TNF-*α* in the peritoneal fluid of mice 1 hour after injection. MT-II, a Lys49, induced a small increase in this parameter only after 6 hours of incubation. Increased production of cytokines, particularly TNF-*α*, can be regulated by increased production of reactive oxygen species in a mechanism dependent on NF-kB. Zuliani et al. [12] demonstrated the ability of these isolated myotoxins to induce a significant increase in H_2_O_2_ production, suggesting that high levels of this reactive oxygen may play an important role in regulating the production of TNF-*α*.

It is important to note that TNF-*α* binds to its cellular TNF-*α* receptor 1 (TNFR1), which triggers signaling cascades that activate NF-*κ*B and AP-1 transcription factors [53, 54]. This cytokine has also been reported to increase reactive oxygen species (ROS) production from mitochondria, plasma-membrane NADPH oxidase and lipoxygenase [55–58]. It is possible that BaTX-II and BaPLA_2_ stimulate ROS production through TNF-*α* production probably through the engagement of a subset of Toll-like receptors (TLR1, TLR2, and TLR4) different from BaTX-I that stimulates ROS through phagocytosis.

Taken together, our data indicate that the all studied PLA_2_s do not interfere with adhesion or detachment of J774A.1 macrophages. BaTX-I, but not BaTX-II and BaPLA_2_, stimulates opsonized zymosan phagocytosis via a complement receptor. However, all of the PLA_2_s stimulated an increase in superoxide production and lysosome volume by J774A.1 macrophages after 30 min of incubation. Moreover, BaTX-I, BaTX-II, and BaPLA_2_ increases the number of lipid bodies on macrophages submitted to phagocytosis and not submitted to phagocytosis. Only, BaTX-II and BaPLA_2_ induced the release of TNF-*α* by J774A.1 macrophages. It is important to note that PLA_2_ activity is not essential for triggering these effects. Certain regions of the toxin molecules, other than the catalytic site, can interact with cell membranes, leading to the activation of macrophages. These studies have added knowledge about the mechanisms of action of PLA_2_s isolated from *B*. *atrox*, contributing to better characterization of macrophage function.

Regarding the acidic or basic character of these enzymes, there is no evident difference in the activity of phospholipases used in this study. Many of the PLA_2_s isolated from *Bothrops* venoms are basic proteins. Acidic PLA_2_s are still being studied, but the literature shows that these enzymes can induce various pharmacological effects such as platelet aggregation and hypotension. Acidic phospholipases A_2_, in general, are not myotoxic, but this effect may appear on a smaller scale compared to basic phospholipases, which show intense myotoxicity. However, acidic phospholipases have higher catalytic activity [59]. Additional studies with these enzymes, both acidic and basic, are still needed so that we can determine a possible correlation between toxicity and their pharmacological potential.
